# Molecular mechanism and therapy application of necrosis during myocardial injury

**DOI:** 10.1111/jcmm.13575

**Published:** 2018-03-01

**Authors:** Tao Xu, Wei Ding, Muhammad Akram Tariq, Yu Wang, Qinggong Wan, Mengyang Li, Jianxun Wang

**Affiliations:** ^1^ Center for Regenerative Medicine Institute for Translational Medicine Qingdao University Qingdao China; ^2^ Department of comprehensive internal medicine Affiliated Hospital Qingdao University Qingdao China; ^3^ School of Basic Medical Sciences Qingdao University Qingdao China

**Keywords:** death receptor pathway, heart diseases, mitochondrial pathway, programmed necrosis, therapy application

## Abstract

Necrosis is an ancient topic which gains new attraction in the research area these years. There is no doubt that some necrosis can be regulated by genetic manipulation other than an accidental cell death resulting from physical or chemical stimuli. Recent advances in the molecular mechanism underlying the programmed necrosis show a fine regulation network which indicates new therapy targets in human diseases. Heart diseases seriously endanger our health and have high fatality rates in the patients. Cell death of cardiac myocytes is believed to be critical in the pathogenesis of heart diseases. Although necrosis is likely to play a more important role in cardiac cell death than apoptosis, apoptosis has been paid much attention in the past 30 years because it used to be considered as the only form of programmed cell death. However, recent findings of programmed necrosis and the related signalling pathways have broadened our horizon in the field of programmed cell death and promote new pharmacological application in the treatment of heart diseases. In this review, we summarize the advanced progress in these signalling pathways and discuss the pathos‐physiological relevance and therapeutic implication of targeting necrosis in heart diseases treatment.

## 1 INTRODUCTION

Numerous studies show that cardiac cell death is a major cause of heart diseases. Apoptosis and necrosis are 2 major modes of cardiac cell death and largely occurred in the pathogenesis process of heart diseases.^1^ In the past 30 years, apoptosis has attracted most interests because it can be controlled and regulated.^2^, ^3^ Necrosis has been recognized for more than a century and is also evolutionarily ancient.^4^ But necrosis was viewed as a merely accidental cell death mostly resulting from severe physical damage or intense chemical stimulation,^5^ which limited its interests within the research area for a long time.^6^ However, over the later 10 years, these concepts have been challenged and the view of programmed necrosis has drawn most attention when several lines of investigation show that necrosis can be controlled by genetic manipulation or pharmacological interventions. In 2005, the concept that necrosis might also occur in a regulated fashion was finally confirmed when researchers found molecules that inhibited receptor‐interacting protein kinase 1 (RIP1) can protect cells from necrosis.^7^ Further study demonstrated the important role of Nec‐1 in preventing adverse cardiac remodelling after myocardial ischaemia‐reperfusion by necrosis inhibition through targeting RIP1^8^, ^9^ The discovery of kinase RIP3 and RIP1/RIP3 complex formation in necrosis induction had definitely determinate the concept of signalling‐regulated necrosis or necroptosis.^10^, ^11^ This accumulated knowledge has broadly established the concept of programmed necrosis and attracted more passion on the mechanism study that controlled and executed it. And the recognition that not only apoptosis, but also necrosis, may be actively regulated has also redefined the role of targeting necrosis in heart diseases therapy.^12^, ^13^ However, we cannot distinguish the regulated necrosis and passive necrosis. To what extent necrosis can be regulated and how to regulate necrosis are worthy of further study and the understanding of the molecular mechanisms regulating necrosis will help to develop the antinecrosis strategy which has extremely important significance in the heart diseases treatment.

## 2 DEATH RECEPTOR PATHWAY OF NECROSIS

### 2.1 Initiation of death receptor‐mediated necrosis

**Figure 1:**
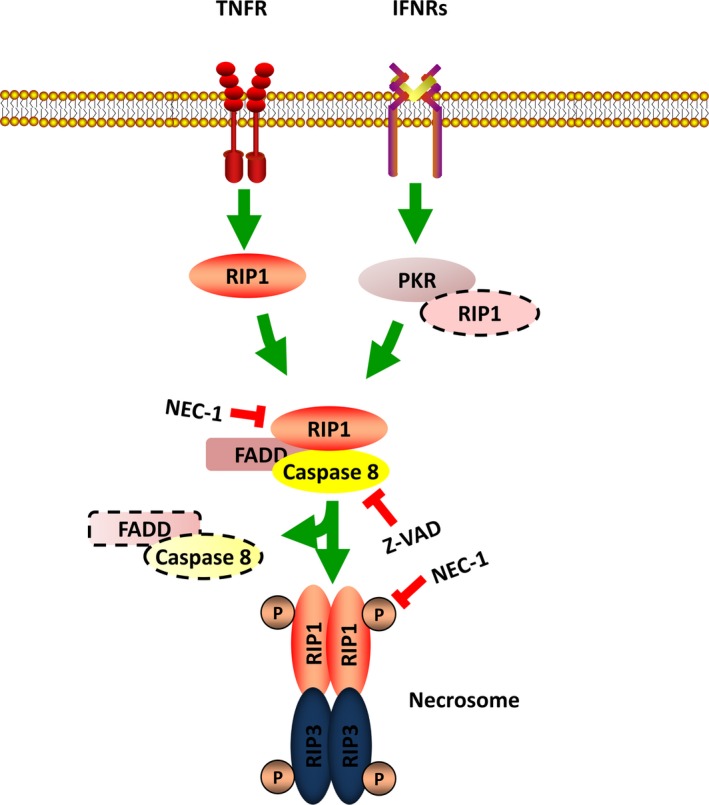
The RIP1‐dependent necrosis pathway. In TNF‐a and IFNRs mediated necrosis pathway, RIP1 is activated and forms necrosome complex with RIP3 when caspase 8 is inhibited. The recruitment of RIP1 by IFNRs needs the activation of PKR which interacts with RIP1

**Figure 2:**
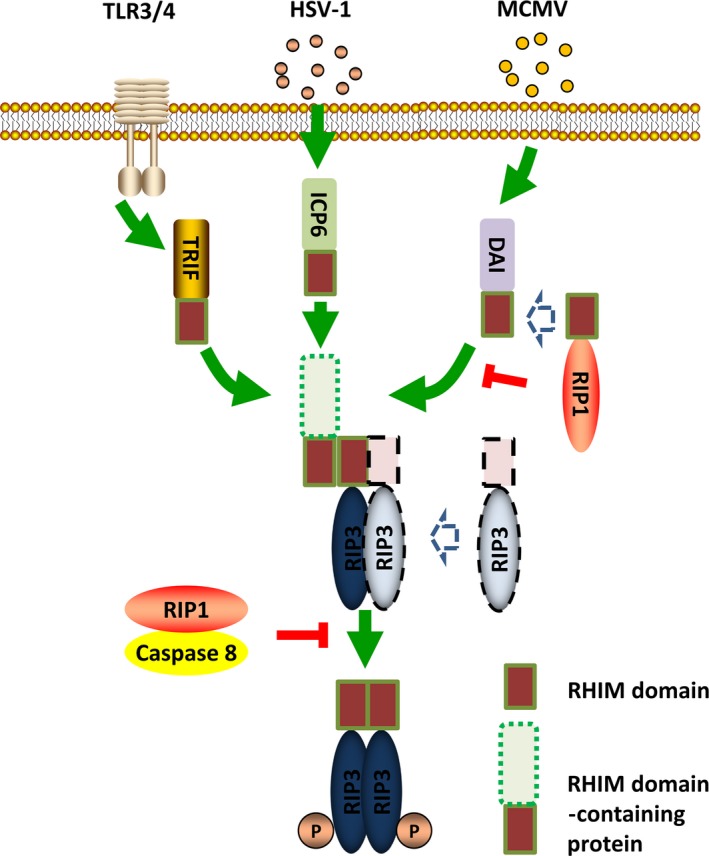
The RIP1‐independent necrosis pathway. In TLR3/4‐, HSV‐1‐ and MCMV‐mediated necrosis, the RHIM domain‐containing protein can interact with RIP3 directly through RHIM‐RHIM interaction. In that case, RIP1 counteracts necrosis by interrupting this interaction. MCMV, murine cytomegalovirus

Necrosis can be induced by various initiators, and the death receptor‐mediated necrosis is the well‐established pathway. When treated with TNF‐a, necrosis will be strongly induced in the case of apoptosis inhibition. The receptor‐interacting protein (RIP) kinases play an important role in the death receptor pathway of necrosis. When activated by TNFR, RIP1 forms a complex with RIP3 and lead to the subsequent activation of RIP3. The RIP1/RIP3 complex is the core regulatory machine called necrosome (Figure 1). With the study going further, several different factors that induce RIP3‐dependent necrosis have been found. Interferons (IFNs) can trigger RIP1/RIP3 complex‐dependent necrosis through transcriptional activation of the RNA responsive protein kinase PKR (Figure 1).^14^, ^15^ RIP1 is needed under some circumstances but not in others. Toll‐like receptors 3 and 4 can directly activate RIP3 via TRIF, a RHIM‐containing protein.^16^ Moreover, RIP1 may even antagonize necrosis by activating caspase 8, the strong inhibitor of RIP3‐mediated necrosis signalling. Another RHIM‐containing protein, DNA‐dependent activator of interferon regulatory factors, DAI, also known as ZBP1, can also activate necrosis through RIP3 activation by the RHIM‐RHIM interaction when cells were infected with murine cytomegalovirus (MCMV).^17^ RIP1 counteracts ZBP1/DAI‐mediated necrosis by interrupting the interaction between ZBP1/DAI and RIP3 in an RHIM domain‐dependent manner which is particularly important for the maintenance of skin homeostasis during late embryonic life and in adult mice.^18^ Necrosis can inhibit HSV‐1 replication in the host cells by HSV‐1 protein ICP6. ICP6 forms dimers/oligomers and then recruits RIP1/RIP3 or RIP3/RIP3 through the direct interaction with RIP1 and RIP3 by its RHIM domain to initiate a necrotic process. HSV‐1 infection is markedly elevated after RIP3 deletion. (Figure 2).^19^, ^20^

### 2.2 Execution of death receptor‐mediated necrosis

It is now clear that RIP3 activation is the key initiation step for the death receptor‐mediated necrosis. But how necrosis is executed after initiation is the focused area of this frontier research now. To this end, several downstream targets of RIP3 have been found and these findings dramatically enrich the knowledge of this signalling pathway.

#### 2.2.1 MLKL

**Figure 3:**
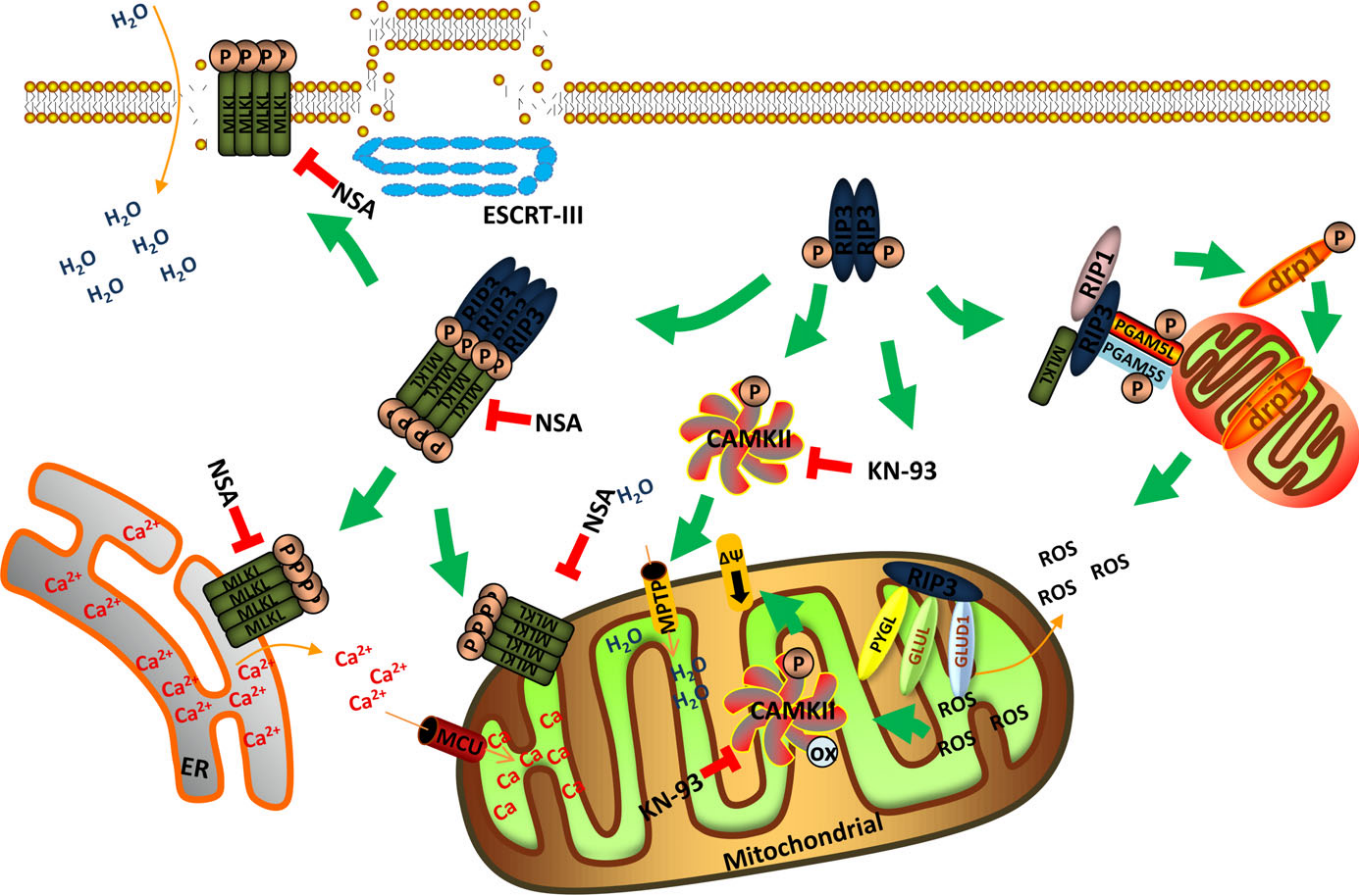
Downstream signalling of RIP3 and necrosis execution. RIP3 phosphorylates MLKL and promotes the formation of MLKL oligomerization. MLKL can target different kinds of membranes including plasma membrane, ER membrane and mitochondrial membrane and lead to membrane disruption. ESCRT‐III preserves membrane intact and contributes to survival. RIP3 promotes mitochondrial metabolism and ROS production by activating PYGL, GLUL and GLUD1. RIP3 promotes MPTP opening and ΔΨ decrease by activating CaMKII activation. The RIP3‐PGAM5‐Drp1 cascade is supposed to be the signalling convergence for necrosis by promoting mitochondrial fission and ROS production

Mixed‐lineage kinase domain‐like protein (MLKL) is the firstly identified RIP3 substrate and the natural target of RIP3 which was found to be specifically required for the RIP3‐dependent necrosis.^21^, ^22^ When necrosis is induced, activated RIP3 binds to MLKL and subsequently phosphorylates MLKL.^21^ Phosphorylated MLKL can disturb the intact structure of the plasma membranes as well as the organelle membrane and change its permeability.^10^, ^23^, ^24^ The N‐terminal coil‐coil domain of MLKL possesses series of amino acids with positive charges and enables MLKL to interact with the negative‐charged phospholipids of the plasma membrane as well as the different phosphatidylinositol phosphates within organelle membranes (Figure 3). NSA is an unique inhibitor of MLKL and disturbs the oligomerization of MLKL and the subsequent necrosis execution.^21^ However, NSA can only target human MLKL which limits its functional validation in other models (Figure 2).

Recently, an interesting work by Yi‐Nan Gong et al implies that cells undergoing necrosis are not always headed towards death: ESCRT‐III helps preserve the plasma membrane in these cells, contributing to survival. They found that cells that exposed PS upon MLKL activation could be “resuscitated” and survive when MLKL activation was subsequently halted, and this required the function of ESCRT proteins belonging to several subgroups (ESCRT‐I, ESCRT‐II and ESCRT‐III) that function in endosomal trafficking, multivesicular body formation, scission of the plasma membrane, virus budding and nuclear membrane assembly during cytokinesis and in the repair of plasma membranes damaged by laser light. Their findings suggest that membrane damage caused by the activity of MLKL during necrosis can be repaired by the function of ESCRT‐III, and thus, cells harbouring active MLKL can sustain survival if ESCRT is engaged (Figure 3).^25^

#### 2.2.2 The metabolic enzymes

The metabolic pathway is actively participant in the execution of necrosis. Several metabolic enzymes including the glycogen phosphorylase (PYGL), glutamate‐ammonia ligase (GLUL) and glutamate dehydrogenase 1 (GLUD1) are found to be the downstream target of the RIP1/RIP3 necrosome. Increased interactions between endogenous RIP3 with PYGL, GLUL and GLUD1 were detected when necrosis was induced (Figure 3).^26^, ^27^ The metabolic enzymes promote the generation of ROS via increasing metabolism and depletion of PYGL, GLUL and GLUD1 by means of siRNA reduced the TNF‐a‐induced accumulation of ROS.^26^, ^27^ And RIP3 might be the activator of these 3 metabolic enzymes according to the results of the in vitro assay. PYGL catalysed the glycogen degradation process. The incubation of purified PYGL and RIP3 showed that RIP3 could enhance its activity but not RIP3‐K51A, which was mutated in the kinase domain. GLUL and GLUD1 function in the Glu or Gln metabolism process during oxidative phosphorylation. Deletion of either GLUL or GLUD1 by RNA interference could also protect cells form TNF+zVAD‐induced necrosis. RIP3 could enhance the activity of GLUL and GLUD1 in vivo, but the direct in vitro activity needs further detection. Thus, RIP3 might promote necrosis by regulating the metabolic enzymes which enhanced energy metabolism and increased production of ROS (Figure 3).

#### 2.2.3 PGAM 5 and Drp1

The mitochondrial protein phosphatase PGAM5 was also reported to be involved in RIP1/RIP3 protein complexes (Figure 3).^28^ PGAM5 presents as 2 splice variants PGAM5L (long form) and PGAM5S (short form). In this work, knockdown of either PGAM5L or PGAM5S could inhibit necrosis induced by TNF‐a, ROS and calcium overload while knockdown of MLKL blocked only TNF‐a‐mediated necrosis. For that reason, PGAM5 was defined as the signalling node for multiple necrosis pathways. PGAM5S but not PGAM5L activated the mitochondrial fission factor Drp1 by recruiting Drp1 and dephosphorylating the serine 637 site of Drp1. Activation of Drp1 caused mitochondrial fragmentation, which promoted ROS production and necrosis execution (Figure 3). In another study, RIP1/RIP3 was reported to directly activate Drp1.^29^ RNA viruses infection initiated assembly of the RIP1‐RIP3 complex, which also activated the GTPase Drp1 and promoted mitochondrial damage and production of ROS. But the role of the mitochondrial PGAM5‐Drp1 axis could not be carried out in some cell lines by several recent studies.^30^, ^31^, ^32^, ^33^ In that case, further research is still needed to validate the function of PGAM5 in different cell lines.

### 2.3 Regulation of death receptor‐mediated necrosis by the post‐translational modification

The post‐translational modification of the RIP1/RIP3 necrosome in the death receptor‐mediated necrosis plays an important role in the necrosis induction and execution. Some involved molecules have been found but a lot more remain unknown.

#### 2.3.1 Ppm1b

Protein phosphatase 1b (Ppm1b) but not protein phosphatase 1a (Ppm1a) is a RIP3 phosphatase. Recent evidence showed that Ppm1b could inhibit both necrosis caused by RIP3 auto‐phosphorylation and necrosis induced by TNF‐a.^34^ Ppm1b could dephosphorylate RIP3 and subsequently prevent recruitment of MLKL to the RIP3 when necrosis was induced in the cells. In the ppm1b deficiency mice, necrosis was enhanced in a RIP3‐dependent manner and elevated RIP3 phosphorylation was also observed when treated with TNF‐a which confirmed the role of ppm1b in necrosis inhibition in the animal model.

#### 2.3.2 Chaperone protein

HSP90 is a molecular chaperone which modulates both the structure and function of its associated proteins.^35^ It has been found that numerous kinases are the targets of HSP90, and these proteins form complexes with HSP90 and its co‐chaperone CDC37.^36^ RIP1 had been demonstrated to be one of the targets of HSP90.^37^ The disruption of interaction between HSP90 and RIP1 resulted in the proteasome‐dependent degradation of RIP1 and the subsequent block of TNF‐a‐induced necrosis.^38^, ^39^ Two new studies by Jacobsen et al and Zhao et al along with recent work by Li et al revealed that HSP90 regulated the stability and function of RIP3 and MLKL.^40^, ^41^, ^42^, ^43^ Recently, Li et al identified RIP3 as the target of HSP90 and revealed the essential role of HSP90 together with its co‐chaperone CDC37 in RIP3 activation. 17AAG, inhibitor of HSP90, disrupted the interaction of RIP3 with HSP90 and blocked the formation of RIP1/RIP3 necrosome formation. But this interaction was not affected by CDC37 knockdown. Furthermore, both inhibition of HSP90 and knockdown of CDC37 blocked the formation of the phosphorylation of RIP3, RIP1/RIP3 necrosome and necrosis. What is more, polymerized RIP3‐induced necrosis was also efficiently blocked by disruption of HSP90 function. Therefore, chaperone protein HSP90 is also an active regulator in necrosis by directly modulating activity of RIP1 and RIP3. The findings of HSP90 inhibitors can develop novel strategy for the treatment of necrosis‐related diseases.

#### 2.3.3 CHIP

CHIP (carboxyl terminus of HSP70‐interacting protein; also known as STUB1) is a chaperone protein and has long been described as an apoptosis inhibitor by degrading a variety of tumour‐suppressive proteins. Recent work showed that CHIP ablation generally accelerated TNF‐a‐mediated necrosis.^44^ CHIP knockout mice showed post‐natal lethality which was rescued by simultaneous knockout of RIP3. In the mechanism study, CHIP could promote the lysosomal localization and subsequent degradation of RIP1 or RIP3 in an ubiquitin‐dependent manner. HSP90 was also closely associated with CHIP and modulated its function. But HSP90 induced the destabilization of RIP1 and RIP3 independent of CHIP as the inhibitor of HSP90 induced the degradation of RIP1 and RIP3 even under CHIP‐depleted conditions.

## 3 MITOCHONDRIAL PATHWAY OF NECROSIS

Mitochondrial permeability transition pore (MPTP) is a multiple protein complex modulating the opening of the inner mitochondrial membrane and allowing the passage of solutes with molecular weights <1500 Da.^45^ MPTP opening leads to the loss of ionic homeostasis, depleted ATP and ultimate necrotic cell death.^46^ The molecular composition of MPTP is elusive probably because the MPTP is a highly dynamic complex.,^47^, ^48^ Cypd is a PPIase and is the key component of the MPTP complex whose activity can promote the opening of MPTP. Cypd seems to be crucial for MPTP−induced cell death as deficiency in Cypd protects neuron, cardiomyocytes and kidney in mice from ischaemia injury. Deficiency in Cypd protected MEF from necrosis induced by calcium overload or H_2_O_2_ treatment.^49^ Cypd−mediated necrosis is also observed in the context of I/R linked pathologies as deficiency in Cypd will dramatically reduced the infarct size.^49^, ^50^ Inhibitors of Cypd, cyclosporine A or sanglifehrin A can inhibit the opening of MPTP and ischaemia injury by preventing the MPTP from opening which provides a certain degree of protection to patients following myocardial infarction (Table 1).^51^, ^52^

**Table 1:** Potential regulators of mitochondrial permeability transition pore (MPTP)

Effect on MPTP	Molecules and factors	Functional roles	Reference
MPTP inhibitors	Cyclosporin A	Specific inhibitors of Cypd; binds and inhibits PPIase activity of Cypd	82, 83
	Sanglifehrin A	Specific inhibitors of Cypd; binds and inhibits PPIase activity of Cypd with different sites from cyclosporin A	51, 52
	Stat 3	Interaction with Cypd	83
	Sirt 3	Interacts with and deacetylates Cypd at lysine 166	82
	HAX1	Interacts with and dissociates Cypd from HSP 90; causes Cypd degradation	81
MPTP stimulators	Ca^2+^	Promote Cypd conformation changes and binding to the IMM	80
	ROS	Promote Cypd conformation changes and binding to the IMM	80
	p53	Direct binding to Cypd and promote Cypd conformation changes	53

The inducers of MPTP seem to involve Ca^2+^, ROS and other factors (Table 1). Recently, p53 was proposed to be the strong inducer of MPTP and regulated MPTP−mediated necrosis through direct interaction with Cypd (Table 1). Binding of p53 would change the conformation of MPTP complex and promoted MPTP opening, but the precise mechanism was still unknown.^53^

Bax and Bak are well known for the regulation of apoptosis. Bax and Bak can be inserted into outer membrane of mitochondrial and are crucial to the outer membrane permeabilization and the cytochrome c release. Recent study by Karch et al showed that Bax and Bak could also regulate the opening of MPTP and necrosis as the outer membrane component of MPTP.^54^ In another work by Whelan RS, they showed that Bax potentiated MPTP opening by promoting mitochondrial fusion which was not dependent on its oligomeric ability.^55^ Thus, Bax and Bak are widely involved in different cell death process and may be therapeutic targets for diseases involved both apoptosis and necrosis.

## 4 SIGNALLING INTERACTION

Now we know that both the death receptor pathway and the mitochondrial pathway are important in the induction of necrosis. But it is currently unclear whether there is one common pathway in which these 2 necrotic pathways converge.

**Figure 4:**
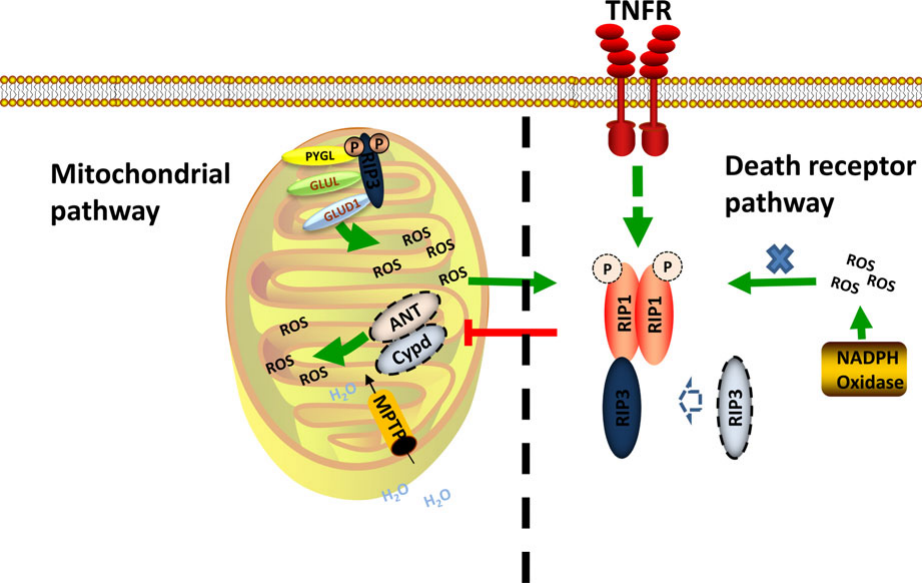
Signalling interaction of the death receptor pathway and mitochondrial pathway. In the death receptor pathway, TNFR can interrupt the interaction of Cypd and ANT in a RIP1‐dependent manner, which produces much mitochondrial ROS. The mitochondrial ROS can promote the auto‐phosphorylation of RIP1 and assembly of the necrosome while the cytoplasm ROS cannot. RIP3 can promote mitochondrial ROS production through activation of the metabolism enzymes. The mitochondrial ROS is also the key stimulator of the MPTP opening

Lots of evidence confirmed that mitochondrial was important in the death receptor‐mediated necrosis. It was commonly proposed that necrotic signalling proceeded through the TNF‐α‐induced accumulation of ROS. TNF‐α‐mediated ROS generation had been shown to be dependent on mitochondrial in L929 cells (Figure 4).^56^ With suppression of complex I of the respiratory chain, TNF‐α‐mediated cell death was attenuated. But inhibiting cytoplasmic ROS generation through knockdown of NADPH oxidase by siRNA did not affect TNF‐α‐mediated cell death (Figure 4). Moreover, recently, Yingying Zhang et al found that mitochondria‐produced reactive oxygen species (ROS) could lead to RIP1 auto‐phosphorylation and was essential for RIP3 recruitment into necrosome.^15^ It was demonstrated that TNF‐a was able to reduce the interaction between ANT and Cypd and impaired the function of ANT in a RIP1‐dependent manner.^57^, ^58^ The direct consequence of impaired function of ANT was the increasing ROS production and decreasing ATP production. Further evidence for the interaction of the 2 pathways was also documented that TNFα‐induced necrosis was attenuated in the Cypd deficiency MEF.^10^ Interestingly, the presence of Nec‐1, a specific RIP1 inhibitor, also delayed opening of the MPTP in isolated cardiomyocytes subjected to oxidative stress.^59^ RIP3 co‐localized with mitochondria when necrosis was induced in a kinase activity‐dependent manner and promoted mitochondrial metabolism and ROS production by activating the PYGL, GLUL and GLUD1, leading to the consequent necrosis as mentioned above (Figures 3 and 4).^60^ In a recent study, Ting Zhang et al found that siRNA‐mediated knockdown of Cypd markedly alleviated RIP3‐induced cardiomyocyte necrosis as assessed by cellular ATP content and LDH release, further revealing the close relationship between these 2 pathways.^61^

However, several recent studies have questioned the role of mitochondrial in death receptor‐mediated necrosis and suggest that mitochondria may be dispensable for this process. Recent work by Andreas Linkermann showed that protection of RIP3 knockout mice was significantly stronger than Cypd knockout mice and Cypd‐RIP3 double‐deficient mice survived prolonged time after kidney I/R injury.^62^ The direct evidence that mitochondria was likely to be dispensable for necrosis was the recent work by Doug Green's group. They cleared the mitochondria of SVEC and 3T3 cells via mitophagy induced by the uncoupler carbonylcyanide m‐chlorophenylhydrazone (CCCP) and demonstrated that TNFα‐induced ROS was lost in these cells, but necrosis was still largely induced.^31^ However, mitochondria could not be completely eliminated with about 20% of cells still contained some mitochondria and still contributed to the necrosis programme. Or maybe, the mitochondrial ROS was just one of the necrosis induction factor and the TNF‐a‐induced necrosis could still be executed without mitochondrial (Figure 4).

In summary, according to the existing evidence, the death receptor pathway can induce necrosis through the mitochondrial ROS and do have some interactions with the mitochondrial pathway but this seems not necessary. They can also function independently and any of the 2 pathways can regulate the necrosis separately. Further study is still needed to reveal the clear relationship between these 2 pathways.

## 5 NECROSIS IN HEART DISEASES

Cell death of the terminally differentiated cardiomyocytes is the major inducer of heart diseases.^63^ Inhibition of apoptosis has been applied to the protection of cardiomyocytes in heart diseases.^64^, ^65^ But the inhibition of apoptosis by caspase inhibitors during early reperfusion did not improve the post‐ischaemic heart function.^66^ During the heart I/R injury, both apoptosis and necrosis would happen, but Leist and his coworkers showed that loss of intracellular ATP would switch the apoptosis to necrosis. The contribution of necrosis to infarct size at 20, 25 and 30 minutes was significantly greater than that of apoptosis. Necrotic disruption of the membranes leads to release of cellular content into the extracellular space and promotes inflammatory reaction and further cell death which is not the case of apoptosis.^67^ Necrosis also plays an important role in heart failure. The frequency of necrosis in heart failure models has been demonstrated early in 1999. Guerra S used the molecular probe to measure the ratio of cardiomyocyte necrosis and apoptosis in the heart failure patients including 7 women and 12 men. In this study, they found that necrosis will cause the DNA damage with blunt end fragments, whereas apoptosis would happen following the double‐strand DNA cleavage with single base or longer 3’ overhangs. The final results showed that heart failure resulted in a great increase in necrosis in both women and men, and levels of cardiomyocyte necrosis were sevenfold greater than apoptosis in the heart failure patients We had no idea how to handle necrosis as it used to be considered a passive and accidental cell death before. Now, the well‐established concept of programmed necrosis and its critical role in the pathogenesis of myocardial infarction, ischaemia/reperfusion injury and heart failure shed light on potential ways for treatment of heart diseases by targeting the necrotic pathways (Table 2).^68^

**Table 2:** Evidence of necrosis in heart diseases

Resources	Manipulation	Observations	Reference
Heart failure patients	Detection of different forms of DNA damages caused by apoptosis and necrosis separately	13‐fold and 27‐fold increase in necrosis in women and men, respectively	72
Heart failure patients	Detection of the level of death receptor	TNF‐a or Fas ligands are elevated in the serum of patients	76, 77
Heart failure patients	Detection of the necrotic markers (pMLKL)	Up‐regulation in the end stage of failing hearts	78
Mouse model	Overexpression of the L‐type Ca^2+^ channel and Ca^2+^ overload	Enhanced sarcolemmal L‐type Ca^2+^ channel (LTCC) activity showed massive myocyte necrosis	^80^
Mouse model	20, 25, 30 min of global ischaemia	Contribution of necrosis to infarct size significantly greater than that of apoptosis	72
Mouse model	CYPD or RIP3 knockout	Markedly decreases infarct size during ischaemia/reperfusion in vivo	^50,61^
Mouse model	Nec‐1 treatment 24 h after induction of ischaemia	Reduction in infarct size	^9,84^

It is believed that necrosis will happen in 2 distinct forms: the passive necrosis and the regulated necrosis or necroptosis. But the relative proportion of unregulated versus regulated necrotic death is not currently known. So to figure out how necrosis process is progressed and which pathways are involved in the regulation of necrosis during the myocardial injury has great significance in the treatment of heart diseases. Recent evidence shows that both the death receptor pathway and the mitochondrial pathway of necrosis are involved in the pathogenesis of heart diseases.

### 5.1 Death receptor‐mediated necrosis in heart diseases

It has long been confirmed that TNF‐a or Fas ligands are elevated in the serum of patients with heart failure.^69^, ^70^ As necrosis can be induced by the activation of the death receptors, it is not surprised that RIP1/RIP3‐mediated death receptor pathway contributes to the necrosis of cardiomyocytes in heart failure patients. In the permanent myocardial ischaemia animal model, RIP3 knockout would decrease the inflammatory response and hypertrophic growth of the cells while the ejection fraction was increased.^60^ It had been also observed that the markers of necrosis, phospho‐MLKL, are also up regulated in the end‐stage heart failure patients.^71^ Until now, several important factors which are involved in the regulation of death receptor‐mediated necrosis in the pathogenesis of heart patients have been revealed.

#### 5.1.1 Tak1 and Traf2

Tak1 was first identified as a transforming growth factor β‐activated kinase, and it was also activated by inflammatory cytokines such as TNF‐α. Tak1 was activated in the heart under the pathological stress such as myocardial infarction in the animal models as well as in patient hearts. Recent work by Lei Li et al showed that Tak1 could regulate TNF‐a‐induced necrosis. Mechanically, activated Tak1 could inhibit the RIP1‐caspase 8‐FADD complex formation and disrupt the downstream signalling of the TNF‐a necrotic pathways through its interaction with RIP1. This interaction was dependent on the kinase activity of Tak1 and could be inhibited by 5Z‐7‐oxozeanol, a selective inhibitor for Tak1. Also, Tak1 could block the formation of RIP1/RIP3 necrosome through FADD and caspase 8. Tak1 was down regulated by severe pressure overload, and Tak1^−/+^ mouse showed greater propensity for cardiac dysfunction and failure after pressure overload stimulation which could be reversed by TNFR1 ablation.^72^ All of this evidence showed a critical cardioprotective role for Tak1 in response to pathological stress. Traf2 was a key component of the TNFR1 signalling complex, which was recruited to TNFR1 by interacting with the adaptor protein TRADD. Recent studies indicated that Traf2 played a protective role in necrotic cell death induced by death ligands. Traf2 could critically regulate polyubiquitination and activation of Tak1 and subsequent inhibition of necrosis.^73^

#### 5.1.2 CaMKII

CaMKII (Ca^2+^‐calmodulin‐dependent protein kinase) was recently found to be another important regulator of necrosis as the targets of RIP3 kinase in heart diseases. During the heart ischaemia/reperfusion injury or doxorubicin treatment, RIP3 induced cardiomyocyte necrosis by CaMKII pathways, rather than the RIP1‐RIP3‐MLKL cascade. CaMKII phosphorylation, oxidation and activity were decreased in RIP3 knockout mouse subjected to ischaemia‐reperfusion injury or doxorubicin. In cultured neonatal myocytes, CaMKII phosphorylation was increased with overexpression of RIP3, and CaMKII inhibition either by dominant‐negative CaMKII or KN‐93, a small molecule CaMKII inhibitor,^74^ decreased cell death in the setting of RIP3 overexpression (Figure 3). Interestingly, RIP3 could directly bind and phosphorylate CaMKII. RIP3 co‐localized with CaMKII in myocytes and a co‐immunoprecipitation assay showed the binding of RIP3 and CaMKII was enhanced in ischaemia‐reperfusion injury or after doxorubicin challenge. Importantly, RIP3 kinase directly phosphorylated recombinant CaMKII protein at the site of T287 in a cell‐free recombinant protein system. The RIP3‐CaMKII cascades were found to lead to the depolarization of mitochondrial inner membrane in a Cypd‐dependent manner. Inhibition of CaMKII blocked mitochondrial depolarization, indicating that MPTP was a critical downstream effector of the RIP3‐CaMKII necrosis pathway. But the targets of the CaMKII on MPTP remained unknown (Figure 3). Taken together, these findings suggested CaMKII served as another RIP3 target and contributed to cardiac necrosis by modulating MPTP. And the inhibition of the RIP3‐CaMKII pathway might be an attractive candidate therapeutic target.^61^

#### 5.1.3 MicroRNA

MicroRNA also plays an important role in the death receptor‐mediated necrosis, and a series of microRNA have been reported to be involved in the necrosis regulation in heart diseases. MicroRNA‐145 could suppress ROS‐induced Ca^2+^ overload of cardiomyocytes by targeting CaMKII. MicroRNA‐155 prevented necrotic cell death in human cardiomyocyte progenitor cells via targeting RIP1.^75^ MicroRNA‐874 could promote myocardial necrosis by targeting caspase 8 which antagonized necrosome formation.^76^ MicorRNA‐873 was found to participate in the regulation of cardiomyocyte necrosis by targeting RIP1/RIP3. In that work, they found that the long noncoding RNA NRF could regulate necrosis by targeting microRNA‐873.^77^ Specifically, Wang found that microRNA‐103/107 regulated programmed necrosis and myocardial ischaemia/reperfusion injury through targeting FADD. In that work, they confirmed that high concentration of H_2_O_2_ would lead to the necrotic cell death while low concentration of H_2_O_2_ would lead to the apoptotic cell death. The expression of microRNA103/107 was dramatically increased when treated with high concentration of H_2_O_2_. MicroRNA‐103/107 induced necrotic cell death by down‐regulating its target FADD. FADD regulated necrotic cell death through influencing the RIP1/RIP3 complex. They also explored the pathogenesis of cardiac infarction in an I/R mouse model and found that miR‐103/miR‐107 were elevated in the ischaemic zone where necrosis largely occurred and the protein levels of FADD in ischaemic zone were decreased. Knockdown of microRNA103/107 shows a protective role during the cardiac I/R injury.

### 5.2 The mitochondrial necrosis pathway in heart diseases

The opening of the MPTP is the critical determinant of necrosis induction and accounts for the major cell death in the first few minutes of reperfusion during cardiac I/R injury, contributing up to 50% of the final infarct size.^78^ Deletion of Cypd markedly decreased MI size during ischaemia/reperfusion in vivo. Similarly, cyclosporine A or sanglifehrin A, which bind and inhibited the activity of Cypd, protected isolated cardiomyocytes from I/R injury and decreased infarction in isolated, perfused hearts and the myocardium in vivo in mouse model. More importantly, cyclosporine A had also been reported to improve the heart function in human. Serum creatine kinase and infarct size were decreased in the cyclosporine A‐treated patients with acute myocardial infarction.^79^ Ca^2+^ overload was the trigger of both MPTP opening and heart failure. Transgenic mice with overexpression of the L‐type Ca^2+^ channel showed myocardial necrosis and heart failure. This phenotype was rescued by the Cypd deficiency but not Bcl‐2 overexpression, indicating necrosis but not apoptosis as the major cause in Ca^2+^ abnormality‐related pathogenesis.^80^ As Cypd is the core regulator of the MPTP opening and necrosis induction in the pathogenesis of heart diseases, factors regulating the activity of Cypd has been studied intensively.

#### 5.2.1 HSP90 and HAX1

The mitochondrial chaperone protein HSP90 present a cardioprotective effect in myocardial damage and can regulate Cypd by protein folding and refolding. HSP90 could interact with Cypd, and the interruption of this interaction would lead to Cypd degradation. The hematopoietic‐substrate‐1 associated protein X‐1 (HAX‐1) could inhibit MPTP opening and necrotic cell death during myocardial damage. HAX‐1 could regulate Cypd protein level by interacting with Cypd and disrupting the interaction of Cypd with HSP90. Overexpression of HAX‐1 would render Cypd prone to ubiquitination and degradation through ubiquitin‐proteasome pathway.^81^

#### 5.2.2 Sirt 3

Sirt 3 is a NAD^+^‐dependent deacetylase and has long been considered as a cardioprotective factor. Recently, Angela V. Hafner reported that Cypd was acetylated on lysine 166 which was adjacent to the CsA binding site. Acetylated Cypd promoted the opening of MPTP in aged cardiomyocytes. Sirt 3 deacetylated Cypd and prevented the MPTP in cardiac tissue during ageing. Sirt 3 knockout mice exhibited a phenotype of age‐related mitochondrial swelling in cardiomyocytes with increased MPTP opening, which was rescued by cyclosporine A. Sirt 3 knockout mice were also prone to cardiac hypertrophy and fibrosis under the stress induced by transverse aortic constriction. Taken together, these data showed that Sirt 3 could deacetylate Cypd and prevent mitochondrial dysfunction and cardiac hypertrophy.^82^

#### 5.2.3 Stat 3

The signal transducer and activator of transcription 3 (Stat 3) had been identified in mitochondrial and contributed to cardioprotection during the ischaemic pre‐ and post‐conditioning. Stat 3 could inhibit MPTP opening by interaction with Cypd. CsA reduced infarct size during ischaemic injury to a similar extent in wild‐type and STAT 3‐KO mice in vivo which indicated the same target between STAT 3 and CsA.^83^

## 6 POTENTIAL USE OF SMALL MOLECULES IN THE TREATMENT OF HEART DISEASES

Many small molecules have been developed to inhibit necrosis and show potential use in the treatment of heart diseases. For the death receptor pathway, interference with necrosis is possible at the necrosis induction, the assembly of the necrosome process and downstream necrosis execution process. The TNF‐a inhibitor has been demonstrated to have no protective role on the pathogenesis of heart diseases, so we focus on the downstream signalling after the activation of death receptor. Nec‐1 was a RIP1 inhibitor that binds with RIP1 specifically and antagonized the activity of RIP1.^8^ Nec‐1 could reduce infarct size markedly during the cardiac ischaemia/reperfusion injury and also prevented adverse cardiac remodelling after ischaemia/reperfusion by blocking the death receptor necrosis pathway in the mouse model.^9^, ^84^ However, the drawbacks of Nec‐1 had been reported during the clinical applicability that Nec‐1 might accelerate cell death in some cases. Fortunately, the second generation of RIP1 inhibitors, Nec‐1s, had been developed and the acceleration of cell death was not observed. Necrosulfonamide (NSA) was another attractive molecules as the direct inhibitor of human MLKL. Although NSA could only target the human MLKL and lack further pharmacokinetics analysis in the mouse models, it provided the cue that MLKL might serve as a potential target. The RIP3 and chaperone protein HSP90 could regulate necrosis as mentioned above. However, both the inhibitors of RIP3 and HSP90 showed an apoptosis effect in some cell lines which limited the use of RIP3 and HSP90 inhibitors in heart diseases treatment. For the mitochondrial pathway, CsA had been applied to decrease the size of the infarct area during acute myocardial infarction in a small number of patients showing a strong potential for clinical treatment. But these results required further confirmation in a larger clinical trial. It had been reported that there was little benefit to combine Nec‐1 with CsA in the clinical application.^84^ However, these conclusions were drawn from only few samples, and it was deserved to re‐evaluate necrosis inhibition by Nec‐1 and cyclosporine A in combination in different cases.

## 7 PERSPECTIVE

Understanding of different modes of cell death due to pathologic stimuli can provide valuable knowledge about the pathophysiology. The recognition that large proportion of necrotic death is regulated aroused great interests in the mechanism study of programmed necrosis. The plentiful and substantial research outcome about programmed necrosis has revolutionized research areas and medicine. But until now, lots of questions remain to be answered. Firstly, how is necrosis signalling transducted? The RIP1/RIP3 complex is the core regulatory factors in necrosis induction, but the downstream signalling is largely unknown. Although some mechanism has been revealed, the more detailed mechanism remains to be further explored. No efficient interfering methods that block necrosis induction can be applied to disease treatment until now. Secondly, what is the molecular connection between necrosis and other death processes? The necrosis and apoptosis share some signalling in the death receptor pathway. Also, some apoptotic factors can also regulate the necrosis process (Bax and Bak) but others cannot (Cypd). Whether necrosis and apoptosis will be initiated separately or both of them will be induced under certain condition needs the development of accurate detection methods that can distinguish apoptosis and necrosis clearly. At last, what is the evolutionary relationship among various forms of cell death? Inhibition of apoptosis will induce the necrosis process under the apoptotic stimuli. The key apoptosis‐inducing factors, FADD and caspase 8, can inhibit necrosis induction. As apoptosis is a very moderate and economic mode of cell death without inflammation, some people even believe that apoptosis is protective for the whole body considering the inhibition of the more harmful necrosis. Recently, autophagy is reported to play roles in the cell death process and some people think necrosis share some feature of autophagic cell death. We know that autophagy happens under normal condition and clears the damaged organelles or proteins. But it is hard to determine whether autophagy can promote or inhibit cell death process under stress. And the role of autophagy in necrosis induction is still under debate. For the future study, it is meaningful to obtain a clear relationship between different modes of cell death pathway and the understanding of what kinds of cell death process will be induced under a certain pathologic stimulus will be clinically helpful.

## DISCLOSURES

All authors declared no conflict of interests.
